# Hydrogel-Nanoparticle Bioactive Platforms for Post-Surgical Prevention of Tumor Recurrence

**DOI:** 10.3390/nano16150924

**Published:** 2026-07-27

**Authors:** Bogdan Mircea Măciuceanu Zărnescu, Denisa Nicoleta Mușat, Adelina-Gabriela Niculescu, Alexandru Scafa Udriște, Alexandru Mihai Grumezescu, Sebastian Vâlcea, Daniela Anghel

**Affiliations:** 1 University of Medicine and Pharmacy “Carol Davila”, 8, Eroii Sanitari, 050474 Bucharest, Romania; bmaciuceanu@gmail.com (B.M.M.Z.); alexandru.scafa@umfcd.ro (A.S.U.); sebastian.valcea@gmail.com (S.V.); 2 Faculty of Chemical Engineering and Biotechnologies, National University of Science and Technology Politehnica Bucharest, Gh. Polizu St. 1–7, 060042 Bucharest, Romania; nicoletamusat9@gmail.com (D.N.M.); adelina.niculescu@upb.ro (A.-G.N.); 3 Research Institute of the University of Bucharest—ICUB, University of Bucharest, 050657 Bucharest, Romania; 4 Department of Internal Medicine 2, Central Military Emergency University Hospital “Dr. Carol Davila”, 010825 Bucharest, Romania; daniela.anghel@prof.utm.ro; 5 Department of Medico Surgical and Prophylactic Disciplines, Titu Maiorescu University, 031593 Bucharest, Romania

**Keywords:** hydrogel-nanoparticle platforms, reccurence, drug release, immunotherapy

## Abstract

One of the main issues in oncology is tumor recurrence following resection, which is responsible for a large number of patient deaths and treatment failures in a variety of malignancies. Despite advancements in adjuvant chemotherapy and radiation, residual disease that remains inside or close to the resection cavity and is difficult for systemic therapies to eradicate is the cause of further local recurrence. Hydrogel-nanoparticle (HNP) composites are a new class of therapeutic platforms that emerged from the recent convergence of biomaterial science and nanomedicine. Their specific goals are to fill the surgical gap, provide long-term localized drug release, and energetically remodel the post-surgical tumor microenvironment (TME). This paper includes the biological foundations of localized post-surgical therapy, important physicochemical considerations of the hydrogel matrix and nanoparticle carrier design, and a compilation of mechanistic and preclinical evidence for HNP hybrid platforms. Immunomodulatory strategies, stimuli-responsive release mechanism engineering, and novel techniques, including combination immunotherapy and 3D-printed customized scaffolds, are all given special attention. Examples of translational challenges are also addressed, such as manufacturing repeatability, biocompatibility, and regulatory classification. When considered collectively, the data demonstrate that HNP platforms are a convincing, practically feasible approach to reducing post-surgical recurrence rates and enhancing patient outcomes.

## 1. Introduction

### 1.1. Clinical Burden of Post-Surgical Tumor Recurrence

Surgical resection is still the main curative modality for most solid tumors, but the risk of local recurrence following seemingly complete excision is significant and differs greatly between tumor types [1,2]. In glioblastoma multiforme (GBM), recurrence rates approach 90%, with the vast majority of events occurring within 2 cm of the original resection margin [3,4]. Breast cancer presents local recurrence rates between 5% and 30% depending on molecular subtype and surgical margins, while colorectal cancer recurs locally in approximately 10–30% of cases despite standard-of-care adjuvant treatment [5,6]. Pancreatic and hepatocellular carcinomas carry particularly poor prognoses, with recurrence rates exceeding 70% and 60%, respectively, within two years of resection [7,8,9].

Central to this phenomenon is the concept of minimal residual disease (MRD), which refers to the persistence of a viable tumor cell subpopulation after macroscopic resection. Such cells may remain dormant for variable periods or resume active proliferation upon exposure to pro-tumorigenic signals generated by the post-surgical wound healing milieu [2,10]. MRD should not be seen simply as an indication of inadequate surgical technique; in fact, margin-negative resections can still contain microscopic tumor foci that can lead to future recurrences. At the molecular scale, MRD includes cells that exhibit stem-like characteristics and are resistant to standard treatment methods, circulating tumor cells, and dispersed cells found in remote organs [7,11].

Local recurrence has clinical consequences that extend well beyond mere oncological results. Recurrence patients experience a quantifiable decrease in overall survival, a gradual deterioration in quality of life, and a significantly higher burden of subsequent therapeutic interventions, such as palliative radiotherapy, systemic chemotherapy, and repeat surgery [12]. Recurrence prevention is one of the highest-yield targets for intervention across the cancer care spectrum from a public health perspective, and it places a significant cost burden on healthcare systems.

The limitations of surgical resection are mostly biological and anatomical in nature. The tumor extends diffuse projections into the surrounding parenchyma in infiltrative malignancies, most commonly glioblastoma, which cannot be safely treated without causing intolerable neurological morbidity [3,13]. The amount of normal tissue that can be lost while maintaining function in other tumor subtypes is limited by the close proximity to important anatomical structures. Beyond these anatomical elements, the surgical procedure itself disrupts immune surveillance, alters local tissue architecture, and initiates a cascade of wound-healing processes that inadvertently create a microenvironment favorable to residual cell proliferation [4,14,15]. When combined, these inherent limitations underscore the clinical need for postoperative adjuvant therapies targeting the local tumor site.

### 1.2. Limitations of Current Adjuvant Therapies

The conventional approach to mitigating post-surgical recurrence across most solid tumor types relies on systemic adjuvant chemotherapy, generally initiated within weeks of the index procedure and frequently combined with radiotherapy. Although these modalities clearly lower recurrence risk in suitably selected patient groups, their effectiveness at the surgical site is limited by intrinsic pharmacokinetic and pharmacodynamic constraints that systemic administration cannot easily overcome [2].

Successful systemic chemotherapy relies on adequate drug distribution to residual tumor areas; however, the levels achievable within a resection cavity are influenced by local blood flow, vascular permeability, and interstitial fluid movement—factors that are significantly altered in post-surgical tissue [16]. Agents given intravenously must pass through the systemic circulation, reach the target site, and diffuse into the extracellular matrix in sufficient amounts to produce a significant cytotoxic effect. In reality, drug levels at the resection margin often turn out to be subtherapeutic, especially in tumors that are poorly vascularized or centrally necrotic [17]. This challenge is further compounded in CNS malignancies by the blood-brain barrier (BBB), which prevents most conventional chemotherapeutic agents from reaching therapeutic concentrations in the brain parenchyma. Temozolomide, the alkylating agent of choice in GBM management, demonstrates acceptable BBB permeability but remains associated with early recurrence, largely due to the emergence of acquired resistance mechanisms [18,19].

Systemic toxicity increases pharmacokinetic limitations, thus decreasing the therapeutic window in which adjuvant chemotherapy can be effectively delivered. Myelosuppression, peripheral neuropathy, nephrotoxicity, and gastrointestinal toxicity are examples of dose-limiting side effects that frequently need dose reductions or treatment interruptions, both of which have the potential to minimize overall therapeutic efficacy [20]. The disconnect between the tumor site and the sites of toxicity, bone marrow, peripheral nerves, and gastrointestinal epithelium, highlights the fundamental inefficiency of systemic delivery when the therapeutic goal is local disease control.

Radiotherapy addresses some of these limitations by directing energy to the target field, but its efficacy is constrained by the radiation tolerance of adjacent normal tissues. In the brain, dose escalation beyond standard fractions is associated with radiation necrosis and neurocognitive decline [19,21]. In the abdomen and pelvis, bowel and bladder toxicity impose dose ceilings. Furthermore, radiotherapy cannot address residual cells that have migrated beyond the radiation field, and hypoxic tumor cells at the resection margin are inherently radioresistant because radiation-induced DNA damage depends on molecular oxygen [22]. Taken together, these limitations define a therapeutic gap that existing systemic modalities cannot bridge, and motivate the development of locally acting platforms capable of delivering sustained, high-concentration therapy precisely where residual disease persists.

### 1.3. Rationale for Localized Bioactive Platforms

The concept of local drug delivery for cancer treatment is not new; the Gliadel wafer, a carmustine-loaded polyanhydride disc implanted into the GBM resection cavity, was approved by the FDA in 1996 and demonstrated modest survival benefits in clinical trials [19,23]. However, Gliadel’s limitations, fixed drug loading, lack of stimuli-responsiveness, poor conformability to irregular cavity geometries, and inability to address the immunosuppressive microenvironment, illustrate precisely the design shortcomings that next-generation bioactive platforms aim to overcome.

The fundamental principle of localized bioactive platforms is the creation of a spatially confined therapeutic depot that maintains effective drug concentrations at the site of residual disease over a clinically meaningful period, typically spanning weeks to months [24]. Limiting drug release to the resection cavity significantly reduces systemic exposure, allowing local concentrations to reach levels that might be harmful if administered intravenously, while also protecting distant organs from unintended side effects. This principle of spatial confinement further extends the repertoire of deliverable agents to include those inherently unsuitable for systemic administration—among them macromolecular biologics, nucleic acid payloads, and immunomodulatory cytokines—whose clinical translation has historically been precluded by poor biodistribution, susceptibility to rapid degradation, or prohibitive systemic toxicity profiles [19,25].

In addition to merely providing cytotoxic agents, emerging bioactive platforms are increasingly designed to actively modify the post-surgical microenvironment rather than solely serving as passive drug-release reservoirs. Surgical procedures initiate a wound-healing response that may inadvertently create conditions conducive to tumor recurrence, including the release of inflammatory cytokines, growth factors that promote cell proliferation, and immune cells with suppressive roles [26]. Collectively, these components can aid in the persistence of remaining tumor cells and their later resurgence. Consequently, an efficient local delivery system must not only supply therapeutic agents but also address fundamental biological processes—such as modulating immune responses, disrupting angiogenesis, and preventing the reestablishment of a microenvironment that fosters tumor development [19,26].

The incorporation of biomaterial platforms into surgical processes also introduces several practical considerations beyond biological and pharmacological factors. In a clinical environment, the substance must be simple to use, conform to the uneven shape of the resection cavity, and polymerize on-site quickly without causing thermal or chemical harm to adjacent tissue. Furthermore, its degradation pattern must correspond with the natural schedule of tissue recovery and remodeling [27]. These engineering specifications are intricately linked to the biological and therapeutic goals of the system, and collectively they establish the framework for developing hydrogel–nanoparticle composite platforms.

## 2. The Post-Surgical Tumor Microenvironment as a Therapeutic Target

### 2.1. Biological Features of the Post-Resection Niche

The cavity created after tumor excision should not be considered a fixed area but rather a dynamic biological environment influenced by surgical injury, local hypoxia, and the initiation of wound-healing mechanisms. These modifications create conditions that may promote tumor recurrence over time. A comprehensive understanding of the changing biology of this post-surgical environment is thus crucial for developing treatment strategies that interrupt the sequence of events that ultimately lead to tumor regrowth [28].

Damage-associated molecular patterns (DAMPs) released by damaged tissue cells trigger an early inflammatory response within hours of surgical resection [28]. Neutrophils and macrophages are drawn in as a result of this reaction, and they release pro-inflammatory cytokines such as TNF-α, IL-1β, IL-6, and IL-8. This early inflammatory activity can have unexpected consequences, even though it is necessary for wound healing and the elimination of cellular debris. Specifically, the resultant cytokine milieu may stimulate signaling pathways that promote the survival and proliferation of remaining tumor cells, such as NF-κB and STAT3 [19,29]. As healing progresses, the transition to the proliferative phase is accompanied by a shift in macrophage phenotype toward an M2-like, tissue-remodeling state. Although crucial for repair, these cells may also promote tumor progression by releasing factors such asGF-β, VEGF, and matrix metalloproteinases (MMPs) [30].

A second key characteristic of the post-resection microenvironment is tissue hypoxia. Interruption of local blood vessels during surgical removal leads to areas of oxygen shortage, which stabilize hypoxia-inducible factor 1-alpha (HIF-1α), a key transcriptional regulator that coordinates the cellular adaptive response to low oxygen conditions [31]. HIF-1α activation drives expression of VEGF and its receptors, initiating an angiogenic program that supplies emergent blood vessels to regenerating tissue, but also supports the metabolic requirements of proliferating residual tumor cells. At the same time, hypoxia-induced oxidative stress produces reactive oxygen species (ROS) that, at sub-lethal levels, stimulate pro-survival kinases such as Akt and ERK in cancer cells [32].

**Figure 1:**
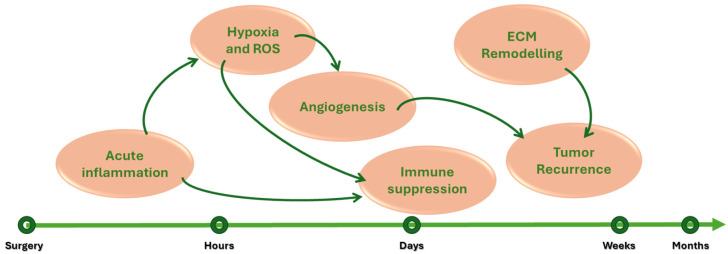
Sequential biological events in the post-surgical TME contributing to the establishment of recurrence [28,29,30,31,32,33,34].

Remodeling of the extracellular matrix is another vital aspect of the post-surgical microenvironment that may play a role in tumor recurrence. Alteration of the normal stromal architecture results in the liberation of growth factors that were previously sequestered, such as TGF-β, FGF, and EGF, which can trigger signaling pathways in remaining tumor cells [33]. At the same time, the formation of a provisional fibrin matrix provides a structural scaffold that supports cell migration and invasion. In parallel, matrix metalloproteinases (MMPs), produced by infiltrating immune cells and activated fibroblasts, degrade components of the basement membrane and facilitate the spread of tumor cells beyond the resection margin [34]. Taken together (Figure 1), these processes—including inflammatory signaling, hypoxia-driven angiogenesis, matrix remodeling, and immunosuppressive cell activity—define the biological context that therapeutic strategies need to address.

### 2.2. Mechanisms Driving Tumor Recurrence

The recurrence of tumor following surgical resection is not driven by a single cell population or a uniform mechanism, but rather by the convergence of multiple complementary processes that collectively enable the outgrowth of residual disease. A mechanistic understanding of these processes is necessary to design platforms that comprehensively address tumor regrowth [35,36].

Cancer stem cells (CSCs) represent a hierarchically defined subpopulation within solid tumors characterized by enhanced self-renewal capacity, pluripotency, and resistance to conventional cytotoxic therapies [35,36]. Following surgical resection, the selective pressure imposed by adjuvant chemotherapy and radiotherapy enriches for CSCs within the residual cell population, as differentiated tumor cells undergo apoptosis. In contrast, CSCs resist treatment through enhanced DNA repair, elevated ABC transporter expression, and a quiescent cell cycle state that reduces exposure to cycle-dependent agents [37]. CSCs participate in two-way communication with the post-surgical niche, reacting to hypoxic and inflammatory cues that enhance their self-renewal and facilitate the restoration of diverse tumor cell populations.

Epithelial–mesenchymal transition (EMT) is an essential process that may lead to tumor recurrence after surgery. Through this process, epithelial cancer cells acquire mesenchymal features, such as reduced cell–cell adhesion, cytoskeletal reorganization, and increased migratory capacity, which allow them to invade surrounding tissues and disseminate [38,39]. The microenvironment after surgery provides various signals that can trigger EMT, including TGF-β, TNF-α, and hypoxia-associated transcription factors. Moreover, cells that experience EMT are often less sensitive to standard chemotherapy and may exhibit stem-like features, connecting this phenomenon to the previously mentioned cancer stem cell (CSC) population.

Tumor dormancy and its eventual disruption represent one of the most challenging aspects of recurrence biology. After apparently successful surgical treatment, residual tumor cells may persist in a quiescent state for extended periods, evading immune detection and resisting therapeutic elimination [40]. This dormant state is maintained by a balance between pro- and anti-proliferative signals, as well as by effective anti-angiogenic control and immune surveillance. However, this balance can be disrupted, including by signals associated with wound healing following surgery, which may reactivate dormant cells and ultimately lead to delayed recurrence. At the same time, tumor cells can employ various immune evasion mechanisms, such as upregulation of PD-L1, reduced MHC-I expression, and the recruitment of regulatory T cells (Tregs) and myeloid-derived suppressor cells (MDSCs), further limiting immune-mediated clearance. In parallel, the development of new blood vessels, driven in part by VEGF produced by tumor cells and tumor-associated macrophages, provides the necessary support for renewed tumor growth [41].

## 3. Hydrogels as Local Therapeutic Depots

### 3.1. Classification of Hydrogels

The ability of hydrogels, three-dimensional polymer networks, to retain and absorb large amounts of water without losing structural integrity makes them ideal for biological applications such as tissue engineering, drug delivery, and wound healing. Hydrogels are outstanding options for therapeutic applications in the post-surgical environment due to their biocompatibility and ability to conform to irregular cavity shapes. Their physical and chemical characteristics, including rigidity, porosity, breakdown rate, and surface chemistry, can be altered to satisfy particular needs [42,43].

**Figure 2:**
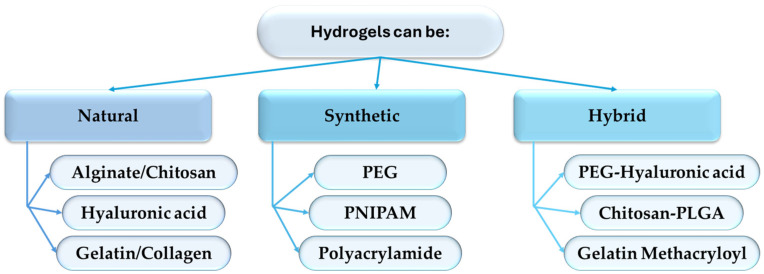
Classification of hydrogel systems used in post-surgical drug delivery applications, according to their chemical composition [42,44,45,46,47,48].

Hydrogels can be categorized in multiple ways. They may be broadly categorized as natural, synthetic, or hybrid systems according to their chemical composition (Figure 2). Natural hydrogels are derived from biological polymers, such as protein-based structures (e.g., gelatin, collagen, and fibrin) and polysaccharides (e.g., alginate, chitosan, hyaluronic acid, and dextran) [44]. These substances are often physiologically active and naturally bio-compatible. For example, hyaluronic acid binds to CD44 receptors on stromal cells and cancer cells, facilitating receptor-mediated interactions in addition to simple drug release. Nonetheless, achieving consistent release profiles can be difficult due to inherent batch-to-batch variability, low mechanical strength, and rapid enzymatic degradation [44].

In contrast, synthetic hydrogels offer precise chemical composition and highly adjustable properties. PEG-based hydrogels represent the most extensively researched synthetic system, appreciated for their minimal immunogenicity, resistance to non-specific protein binding, and ease of functionalization via terminal reactive groups [45]. PNIPAM and its copolymers are the primary category of thermoresponsive synthetic hydrogels, displaying a lower critical solution temperature close to body temperature, enabling the material to switch from liquid to gel following injection. Polyacrylamide and polyvinyl alcohol hydrogels provide strong mechanical properties, but their non-biodegradable characteristics must be thoughtfully evaluated in implantable applications. Hybrid hydrogels combine natural and synthetic components to take advantage of both—PEG-hyaluronic acid conjugates, for example, pair PEG’s chemical stability with hyaluronic acid’s biological activity [46].

The crosslinking approach employed significantly influences the mechanical characteristics and degradation behavior of a hydrogel. Physical crosslinking depends on non-covalent interactions—hydrogen bonds, hydrophobic associations, electrostatic forces, or chain entanglement—to create temporary networks that are generally shear-thinning and capable of self-healing. Chemical crosslinking creates covalent bonds, resulting in robust, stable networks that resist shear forces; however, the need for chemical initiators or UV light can limit their application in surgical settings. The use of enzymatic crosslinking with transglutaminase or peroxidase systems offers a cleaner alternative that operates under physiological conditions, without requiring external initiators [48]. The degradation process—be it hydrolytic, enzymatic, or stimuli-induced—affects the release rate of encapsulated therapies over time and the implant’s resolution speed, ideally aligning with the peak risk of recurrence.

### 3.2. Injectable and In Situ Forming Hydrogels

To be useful in surgical applications, a hydrogel must be injectable—administered in liquid form via a needle or catheter—and able to form a stable gel directly in the resection cavity, adapting to its often uneven shape without requiring accurate manual positioning. This injectability demand has led to significant advancements in stimuli-responsive hydrogels, in which the transition from sol to gel is triggered by specific environmental signals detected after administration [49].

Thermoresponsive hydrogels exploit the phenomenon of inverse solubility: gelation upon heating rather than cooling, characteristic of polymers exhibiting a lower critical solution temperature (LCST). PNIPAM and its derivatives, as well as poloxamer-based systems (Pluronic F127) and methylcellulose, undergo sharp sol-to-gel transitions as temperature rises from ambient to physiological levels. This property enables the material to be prepared and loaded at room temperature, injected in a low-viscosity state through a standard surgical needle, and to rapidly gel upon contact with body-temperature tissue. The primary limitation of thermoresponsive systems is that the LCST must be precisely tuned; too low, and the material gels prematurely during preparation; too high, and gelation fails at physiological temperatures [50].

pH-responsive hydrogels exploit the acidic microenvironment characteristic of post-surgical tissue and, more markedly, of tumors, where anaerobic glycolysis leads to local pH values of 6.5–6.8 compared to systemic pH of 7.4. Polymers bearing carboxylic acid or amine functional groups undergo protonation-state-dependent conformational changes that drive gelation at specific pH thresholds. This pH-responsiveness can be engineered to function bidirectionally, forming a stable gel under mildly acidic tumor microenvironment conditions while releasing its payload upon exposure to the even lower pH generated within the endosomes of tumor cells that have internalized nanoparticles [43,51].

Enzyme-responsive hydrogels represent a more biologically specific category, exploiting the upregulation of specific enzymatic activities in diseased tissue. MMP-cleavable crosslinkers incorporate peptide sequences, most commonly the GPLGIAGQ substrate for MMP-2 and MMP-9, that are cleaved by MMPs upregulated in the post-surgical TME [52]. This confers inherent selectivity to the degradation and drug release process, coupling therapeutic payload delivery to a biological signal specific to the tumor milieu. Self-healing and shear-thinning hydrogels, formed through reversible physical crosslinks, can be loaded into a syringe, injected under shear stress that disrupts the network, and then spontaneously reconstitute after stress removal, a property that minimizes mechanical trauma to the gel and ensures complete cavity filling [53].

### 3.3. Functional Roles in Post-Surgical Applications

Hydrogels employed in post-surgical environments do more than just transport and deliver medications—they fulfill multiple functional roles that can individually aid in preventing recurrence. One of these involves filling space. In tumors where surgery creates a space that slowly fills with cerebrospinal or serous fluid, this fluid-filled environment may serve as a medium for the proliferation of residual tumor cells [54]. A hydrogel that conforms to and occupies this space physically displaces that fluid and reduces the area available for tumor cells to expand.

Hydrogels release drugs through two main mechanisms: diffusion of encapsulated molecules through the hydrogel mesh, and release coupled to matrix degradation. The mesh size, determined by polymer concentration, crosslink density, and swelling ratio, controls how easily drug molecules can move through the material. For small molecules below 1 kDa, diffusion tends to be relatively fast, often requiring nanoparticle encapsulation to extend release beyond a few days. Macromolecules, including proteins, nucleic acids, and nanoparticle-drug conjugates, exhibit substantially greater diffusional resistance, enabling extended release kinetics solely from the hydrogel matrix [55].

The barrier properties of hydrogels against tumor cell migration have been demonstrated in several in vitro and in vivo studies. Dense hydrogel matrices can physically stop the infiltration of residual tumor cells through a steric mechanism, while the incorporation of anti-migratory agents can augment this effect chemically [43,56]. The rate at which a hydrogel degrades must be precisely aligned with the timeline of tissue healing. If the material deteriorates too rapidly, the therapeutic depot is eliminated before the peak recurrence risk has elapsed; if it deteriorates too slowly, it could hinder normal tissue regeneration and provoke chronic inflammation [39,57]. Mechanical characteristics are important as well—a hydrogel that is either excessively rigid or too pliable compared to the adjacent tissue can lead to interfacial stress and inadequate contact with the cavity walls, decreasing the surface area accessible for drug diffusion.

## 4. Nanoparticles for Targeted and Controlled Therapy

### 4.1. Nanoparticle Platforms

**Figure 3:**
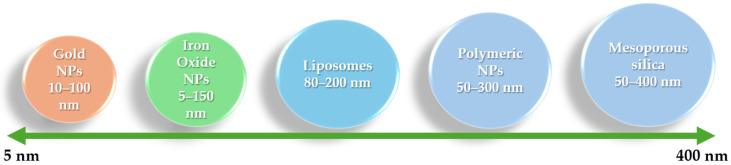
Major nanoparticle platforms used in post-surgical cancer therapy, with representative size ranges [57,62,63,64,65,66,67,68].

Nanoparticles offer a complementary set of properties to hydrogel matrices, addressing the limitations of macroscale polymer systems and enabling functionalities not accessible at the bulk level. Their nanoscale dimensions, typically in the range of 10–400 nm (Figure 3), confer surface-to-volume ratios that facilitate high drug loading, surface functionalization, and interaction with cellular structures, while enabling passive accumulation in tumor tissue through the enhanced permeability and retention (EPR) effect [57,58,59,60,61].

Polymeric nanoparticles represent the most extensively developed class of drug delivery vectors. PLGA (poly(lactic-co-glycolic acid)) nanoparticles are particularly well-studied due to PLGA’s FDA-approved status, biocompatibility, and hydrolytic degradability, which enable sustained drug release profiles ranging from days to months, depending on copolymer composition and particle geometry [39,62]. Chitosan nanoparticles provide inherent cationic charge enabling electrostatic condensation of anionic nucleic acid payloads, and exhibit mucoadhesive properties that prolong residence time at mucosal surfaces. Polymeric micelles formed from amphiphilic block copolymers self-assemble to encapsulate hydrophobic drug molecules within their hydrophobic cores while presenting a hydrophilic PEG corona that reduces opsonization and extends systemic circulation, properties that remain relevant even within locally administered hydrogel composites, thereby determining the fate of nanoparticles released from the gel matrix [63].

Lipid-based nanoparticles, including liposomes and solid lipid nanoparticles (SLNs), constitute the most clinically translated class of nanomedicine vectors, with multiple formulations, including Doxil and Abraxane, achieving regulatory approval [64]. Liposomes consist of a phospholipid bilayer shell enclosing an aqueous core, enabling the encapsulation of both hydrophilic and hydrophobic drugs in the aqueous interior and the lipid bilayer, respectively. Their structural similarity to cell membranes confers inherent biocompatibility and enables membrane fusion-mediated intracellular delivery. Lipid nanoparticles have gained significant clinical validation, most recently as the delivery system for mRNA-based vaccines, where they have demonstrated the ability to protect nucleic acid cargo from enzymatic degradation and facilitate endosomal escape [65].

Inorganic nanoparticles constitute a diverse group, both structurally and functionally, including gold nanoparticles, mesoporous silica nanoparticles (MSNs), iron oxide nanoparticles (IONPs), and calcium phosphate-based systems. Gold nanoparticles are prized for their plasmonic properties, enabling them to convert near-infrared laser energy into targeted heat that can eliminate tumor cells with precise spatial control [59,61,66,69]. MSNs provide exceptionally large surface areas and pore volumes, enabling drug loadings that exceed those of most polymeric systems; their silanol-rich surface chemistry also facilitates the attachment of targeting ligands and stimuli-responsive gatekeeping mechanisms [67]. IONPs are paramagnetic, which makes them useful for MRI contrast enhancement, magnetic hyperthermia, and magnetically guided drug delivery—capabilities particularly relevant for post-surgical monitoring and image-guided therapy. Biomimetic nanoparticles coated with membranes derived from cancer cells, platelets, or red blood cells represent an emerging approach that uses biological camouflage to avoid immune clearance and selectively target residual tumor cells [68,69].

### 4.2. Functional Strategies to Prevent Recurrence

In the post-surgical environment, therapeutic approaches enabled by nanoparticle platforms go far beyond straightforward cytotoxic drug delivery. These strategies include immunomodulation, gene therapy, and energy-mediated tumor ablation, all of which offer a multimodal approach to recurrence prevention.

By separating drug release kinetics from hydrogel degradation, localized chemotherapeutic administration using nanoparticles embedded in hydrogel matrices bypasses the pharmacokinetic limitations of soluble drug formulations. Long-term, continuous distribution of cytotoxic drugs to the site of residual disease is enabled by the secondary nanoparticle-mediated release mechanism [70]. Several preclinical investigations have shown that PLGA nanoparticles loaded with paclitaxel, doxorubicin, or temozolomide and embedded in hydrogel matrices exhibit greater local drug retention and lower systemic toxicity than soluble drug-hydrogel formulations. Also, by protecting labile molecules from enzymatic degradation and altering their intracellular trafficking to get around efflux pump-mediated resistance, the nanoparticle carrier facilitates the solubilization of poorly water-soluble medications [70].

The immunosuppressive nature of the post-surgical TME is directly addressed by immunomodulatory payload administration via nanoparticles. Without the systemic cytokine storm associated with intravenous immunostimulant treatment, nanoparticles loaded with toll-like receptor (TLR) agonists, such as CpG oligonucleotides or poly(I:C), can stimulate local innate immune responses, increasing macrophage and dendritic cell activation [71]. Compared with typical intravenous checkpoint inhibitor dosing, anti-PD-1/PD-L1 antibody-loaded nanoparticles significantly reduce systemic immunotoxicity while enabling local immune checkpoint blockade at doses achievable only by local administration. The administration of cytokines, such as IL-12, IFN-γ, and GM-CSF, via nanoparticles can change the immunological milieu in the resection cavity from an immunosuppressive to an immunostimulatory phenotype.

A rapidly developing area of post-surgical recurrence prevention is gene therapy delivered via nanoparticles. When condensed into cationic or lipid nanoparticles, siRNA targeting oncogenes, pro-survival kinases, or immunosuppressive factors can be administered locally to silence critical targets in remaining tumor cells [72]. With proven effectiveness in several cancer models, CRISPR-Cas9 genome-editing systems encapsulated in lipid or polymeric nanoparticles have been employed preclinically to irreversibly disrupt tumor cell survival genes. Targeting the post-transcriptional networks that control the future direction of tumor cells, microRNA mimics and inhibitors provide extra regulatory power. By combining therapeutic precision with the synergistic benefit of immunogenic cell death that triggers anti-tumor immunity, photothermal and photodynamic therapy, mediated by gold nanoparticles and photosensitizer-loaded formulations, respectively, provide spatially controllable tumor cell ablation that can be externally triggered by near-infrared laser irradiation [73].

## 5. Hydrogel-Nanoparticle Hybrid Platforms: Design and Mechanistic Synergy

### 5.1. Architectural Strategies

The incorporation of nanoparticles into hydrogel matrices to create composite HNP platforms results in architecturally unique structures whose performance is influenced by the particular geometric and chemical relationships between the nanoparticulate and polymeric components, rather than just an additive combination of their individual properties. Different configurations confer distinct pharmacological and mechanistic properties; hence, the design space for HNP structures is diverse [74].

The simplest architectural method is to physically encapsulate pre-formed nanoparticles within a hydrogel matrix to create nanoparticle-loaded hydrogels. In this arrangement, nanoparticles are distributed throughout the polymer solution before crosslinking, and when the hydrogel mesh gels, they become trapped within it. The rate at which nanoparticles are released into surrounding tissue is regulated by the hydrogel matrix, which serves as the major diffusional barrier. The drug enclosed within the nanoparticle subsequently experiences a secondary release phase via its shell. Modifying the hydrogel crosslink density and the composition of the nanoparticle shell enables individual adjustments to the two-phase profile, facilitating release kinetics of unprecedented duration and intricacy [75].

A closer architectural integration is demonstrated by surface-functionalized hydrogel matrices, in which reactive surface groups covalently bind nanoparticles to the polymer core [71]. This anchoring ensures that drug release from the nanoparticle phase occurs locally rather than at distant sites following nanoparticle diffusion away from the implant, thereby preventing premature diffusion of nanoparticles from the matrix. Additionally, the distribution of the nanoparticulate payload within the matrix can be precisely controlled through spatial covalent attachment. This allows for the development of gradient structures that produce spatial concentration profiles that meet certain biological requirements [76].

The nanoparticle-loaded hydrogel principle is structurally inverted in core-shell structures, in which a hydrogel shell coats a nanoparticulate core, modulating release and providing a biocompatible exterior surface [77]. For inorganic nanoparticles, such as gold and iron oxide, whose surfaces would otherwise interact non-specifically with biological components, this architecture is very favorable. Multiple material phases with different functions are incorporated into multilayer and hierarchical composite systems, such as an inner polymeric nanoparticle core containing a cytotoxic drug, encased in a lipid shell with immunomodulatory surface ligands, and embedded in a hyaluronic acid hydrogel functionalized with targeting peptides [78]. The various therapeutic requirements of these systems are reflected in their structural complexity.

The addition of nanoparticles not only confers bioactive functions but also alters the bulk properties of hydrogel networks by serving as additional physical or dynamic crosslinking sites. Nanoparticles can increase the effective crosslinking density through surface chemistry and interactions with polymeric chains, limiting mobility and facilitating the development of compact network structures with smaller pore sizes. These structural changes enhance the storage modulus (G’), yield stress, and improve the overall mechanical stability of the hydrogel matrix. Higher crosslink density typically results in greater viscosity at low shear conditions, enhancing shape accuracy and retention post-implantation. Injectable nanocomposite hydrogels rely on reversible crosslinking mechanisms, enabling temporary network disruption under shear stress and resulting in significant shear thinning behavior and swift structural recovery after the stress is removed. Consequently, despite the higher viscosity associated with nanoparticle inclusion, these hydrogels enable extrusion through narrow needles or surgical catheters while preserving structural integrity, thereby ensuring localized retention post-administration. Recent literature shows that excessive loading with nanoparticles can produce an excessively stiff network that requires high extrusion forces and compromises catheter delivery, while insufficient crosslinking results in poor mechanical stability and rapid dispersion of the material after implantation [79,80,81].

### 5.2. Dual-Level Controlled Release Mechanisms

Compared to systems that use hydrogels alone, hybrid hydrogel-nanoparticle platforms offer greater control over drug delivery by encapsulating therapies within nanocarriers, which are then released from the hydrogel matrix over time. This design minimizes the burst release often associated with conventional hydrogels while improving the stability of sensitive payloads, such as proteins, nucleic acids, and immunomodulators. In contrast to formulations containing only nanoparticles, the hydrogel matrix functions as a confined reservoir that prevents rapid particle diffusion from the operative area, prolongs retention at the resection site, and reduces early elimination via lymphatic drainage or phagocytosis. Continuous local drug exposure, sequential or multi-stage release patterns, improved therapeutic efficacy, and decreased systemic toxicity are thus made possible by the combination of these elements [82,83,84,85].

Drug release happens at the hydrogel level via a mix of degradation-mediated and diffusion-controlled processes [86]. The early release phase of molecularly dissolved small drugs is dominated by Fickian diffusion through the hydrogel mesh, with the rate determined by the drug’s molecular weight, the size of the polymer mesh, and the partition coefficient between the gel’s aqueous interior and the surrounding tissue fluid [87]. The effective mesh size increases as the hydrogel degrades hydrolytically or enzymatically, thereby accelerating the diffusion of larger payloads and ultimately enabling the bulk dissolution of encapsulated nanoparticles into the surrounding tissue. To provide both immediate and long-lasting therapeutic activity, these processes interact to produce release profiles that change from diffusion-limited in the early phase to degradation-driven in later phases [87].

Drug release occurs at the nanoparticle level according to processes unique to the surface design and particle makeup. Polymer swelling, bulk erosion, and surface desorption all contribute to drug release from polymeric nanoparticles [88]. By triggering faster release in response to particular TME signals, stimuli-responsive nanoparticle systems offer an extra level of control. In the oxidative environment typical of the post-surgical niche, ROS-sensitive nanoparticles containing oxidatively cleavable linkers, such as thioketal, phenylboronic acid-ester, and diselenide bonds, release their payload [89,90]. In the somewhat acidic tumor microenvironment, pH-sensitive systems, such as nanoparticles containing acid-labile bonds or polyelectrolyte shells, quickly destabilize. In response to mild hyperthermia induced by gold nanoparticle photothermal activity, thermally triggered liposomes with phase-transition temperatures above 37 °C release their contents, enabling externally controlled, spatially precise drug delivery [91]. By converting the pathogenic signals of the tumor microenvironment into therapeutic triggers, this stimuli-responsive capability achieves a level of biological specificity that passive delivery techniques cannot match.

### 5.3. Integrated Therapeutic Modalities

When several therapeutic modalities are combined into a single composite architecture, the therapeutic potential of HNP platforms is most entirely realized. This allows for synergistic interactions that exceed the combined efficacy of individual treatments administered sequentially or in simple combination. An increasing understanding of the molecular complexity of tumor recurrence and the various redundant mechanisms by which remaining cancer cells avoid eradication is reflected in the design of multimodal HNP systems [92].

The most-studied multimodality HNP approach is chemo-immunotherapy combos. When administered at the proper dosages, cytotoxic medications such as doxorubicin, oxaliplatin, and paclitaxel can cause immunogenic cell death (ICD), a type of tumor cell apoptosis that releases immunostimulatory damage-associated molecular patterns (DAMPs) such as HMGB1, calreticulin, and ATP, transforming the tumor cell into an in situ vaccine [93]. The immunostimulatory effect of chemotherapy-induced ICD is enhanced when combined with local delivery of immune checkpoint inhibitors or TLR agonists within the same HNP composite, thereby activating innate immune effectors. This results in a systemic anti-tumor immune response that extends beyond the treated resection cavity. A qualitative change in the potential impact of local therapy is represented by an abscopal-type effect, which is mediated by activated T cells migrating from the local immunological environment to distant regions [93].

The neovascularization that supports growing tumor cell populations in the post-surgical microenvironment is disrupted by anti-angiogenic therapies targeting VEGF signaling, administered locally via hydrogel-nanoparticle composites [94]. Local anti-angiogenic administration can suppress concentrations within the resection field without systemic exposure, in contrast to systemic anti-VEGF therapy, which is linked to hypertension, thrombosis, and delayed wound healing. A dual approach that simultaneously eradicates residual cells and stops the vascular supply required for their renewal is achieved by combining anti-angiogenic therapy with cytotoxic therapy. By surface-functionalizing nanoparticle carriers with CSC-targeting antibodies or aptamers, it is possible to target cancer stem cells, which express unique surface markers such as CD44, CD133, and EpCAM. This allows selective delivery of differentiation-inducing agents or epigenetic modifiers to the cell population primarily responsible for recurrence [36,95].

### 5.4. Representative Preclinical Models

A significant, increasingly mechanistically coherent body of data supporting clinical translation is provided by preclinical evidence for HNP composite platforms, which spans a wide range of tumor types and experimental conditions. Orthotopic tumor models, in which tumor cells are implanted in the anatomically appropriate site rather than the flank, provide the most clinically predictive assessment of HNP platform efficacy by recapitulating the tumor microenvironment, vascular anatomy, and surgical context relevant to clinical application [96].

Models of surgical resection cavities serve as the most directly applicable preclinical framework. In these studies, animals with tumors undergo partial or total surgical removal of the primary tumor, followed immediately by insertion of the HNP composite at the resection site, with ongoing assessment of recurrence rates and survival. Research involving GBM models with temozolomide-loaded PLGA nanoparticles integrated into hyaluronic acid or PLGA-PEG hydrogels has shown notable decreases in tumor regrowth when compared to systemic temozolomide therapy, with some studies revealing survival enhancements of 60–80% in median survival. Models of post-surgical breast cancer utilizing doxorubicin alongside immune checkpoint inhibitors in HNP platforms have demonstrated a decrease in local recurrence as well as the prevention of distant metastasis via the systemic immune activation mechanisms outlined previously [97].

Assessment of recurrence rates in these models uses bioluminescence imaging to track luciferase-expressing tumor cell burden within the resection field, enabling longitudinal, non-invasive tracking of tumor cell burden [98]. Survival analysis provides the ultimate measure of therapeutic benefit, while histological evaluation of resected tissues at defined endpoints enables characterization of local tissue response, immune infiltrate composition, and residual tumor burden. Immunological evaluation, including flow cytometric analysis of tumor-infiltrating lymphocytes, macrophage phenotyping, and cytokine profiling, has been instrumental in establishing the mechanistic contributions of immunomodulatory HNP platforms and identifying biomarkers of response that could inform clinical patient selection [98].

An overview of recent advancements in this rapidly changing field is presented by summarizing notable preclinical studies from recent years in Table 1, which emphasizes the variety of hydrogel matrices, nanoparticle formulations, therapeutic targets, experimental models, and treatment results.

The studies in Table 1 show how injectable HNP platforms for cancer therapy after surgery have quickly changed. All HNP platforms included in this table were evaluated in clinically relevant post-surgical resection models, reflecting on local therapies designed to prevent postoperative recurrence rather than primary tumor treatment. Although the hydrogel matrices, nanoparticle designs, and therapeutic payloads differ significantly among studies, they all aim to deliver prolonged local treatment within the surgical site while minimizing systemic drug exposure. Significantly, the advantages of these platforms reach far beyond regulated drug delivery. Numerous contemporary systems integrate localized chemotherapy with additional approaches, including catalytic nanomedicine, photothermal therapy, immune modulation, and programmed cell death promotion, to eliminate residual tumor cells and modify the postoperative tumor microenvironment. Consequently, these multifunctional platforms have consistently demonstrated enhanced management of local tumor recurrence in orthotopic and surgical resection models. Various studies have also indicated further therapeutic advantages, such as improved wound healing, reduced postoperative infection rates, and extended survival, underscoring the capability of HNP composites to approach various clinical issues with a singular localized treatment approach [99,100,101,102,103].

## 6. Immunomodulation and Microenvironment Reprogramming

### 6.1. Macrophage Polarization Strategies

The immune landscape of the post-surgical tumor microenvironment is dominated by tumor-associated macrophages (TAMs), which in most solid tumors exhibit an immunosuppressive M2-like phenotype characterized by the expression of anti-inflammatory cytokines, including IL-10, TGF-β, and IL-4, and by downregulation of pro-inflammatory mediators. This polarization state is maintained by microenvironmental signals, including IL-4, IL-13, and M-CSF secreted by tumor cells and stromal components, and is further amplified in the post-surgical context by the wound-healing cytokine milieu. M2 macrophages promote tumor progression through multiple mechanisms, including the secretion of pro-angiogenic factors, the suppression of cytotoxic T cell activity, the deposition of immunosuppressive matrix components, and the direct promotion of tumor cell invasiveness via MMP secretion [104].

**Figure 4:**
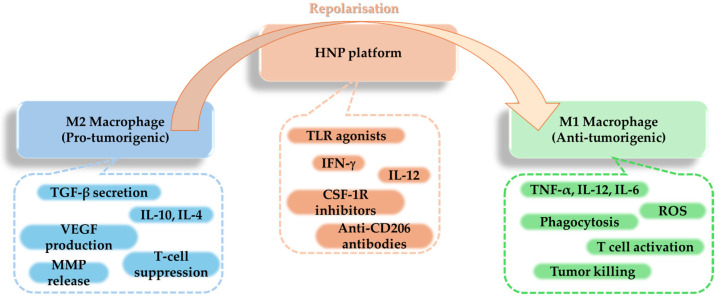
Macrophage polarisation states in the post-surgical TME and the repolarisation strategy enabled by HNP platforms [97,104,105,106].

HNP platforms designed to repolarize TAMs from the M2 toward the pro-inflammatory M1 phenotype have demonstrated impressive preclinical efficacy. Nanoparticles loaded with CSF-1R inhibitors, TLR-4 agonists (LPS or MPLA), or IFN-γ can shift macrophage polarization within the resection cavity, converting immunosuppressive TAMs into tumor-killing effectors [105]. PLGA nanoparticles co-delivering anti-CD206 antibodies (targeting M2 surface markers) and CpG oligonucleotides (TLR-9 agonist), embedded in PEG hydrogels, have demonstrated >70% reduction in the M2:M1 ratio within resection cavities in murine models, accompanied by significant reductions in tumor recurrence. The durability of macrophage repolarization is enhanced when the nanoparticle release is sustained over days to weeks, providing persistent immunostimulatory signaling that prevents the reestablishment of M2 polarization. A schematic visual overview is offered in Figure 4 [105].

### 6.2. Local Immune Checkpoint Inhibition

Immune checkpoint inhibitors targeting the PD-1/PD-L1 and CTLA-4 axes have transformed systemic cancer immunotherapy, but their clinical benefit is accompanied by systemic immune-related adverse events that can be severe and occasionally life-threatening. Local delivery of checkpoint inhibitors via HNP platforms offers a pharmacological strategy to achieve therapeutically relevant concentrations within the immunological microenvironment of the resection cavity while minimizing exposure of systemic immune organs [90,106].

Anti-PD-1 antibody-loaded lipid nanoparticles incorporated into alginate hydrogels have been administered in murine melanoma and colon cancer resection models, achieving local PD-1 blockade with minimal systemic antibody concentrations and substantially reduced immune-related toxicity compared to intravenous injection [97]. The spatial confinement of checkpoint inhibitor delivery to the resection site also concentrates immune activation where tumor antigens are most abundant, at the site of residual tumor cells, rather than diffusely activating systemic T cell populations. This focused activation has been shown to generate more robust tumor-specific immune responses with lower off-target inflammation. Dual checkpoint blockade combining anti-PD-1 and anti-CTLA-4 delivery within a single HNP composite has demonstrated additive benefits in preclinical models, consistent with the known synergy of these agents in the clinical setting, while maintaining a local safety profile superior to systemic combination therapy [97].

### 6.3. Cytokine and Vaccine-Based Approaches

Beyond checkpoint inhibition, local platforms offer unique opportunities to deliver cytokines and tumor antigen-presenting formulations that prime systemic anti-tumor immunity from the local site, effectively converting the resection cavity into an in situ vaccination platform [85]. The principle of this approach exploits the presence of residual tumor antigens at the surgical site, made available by cell death during resection, in combination with locally delivered danger signals and immune stimulants to activate dendritic cells and cross-presenting macrophages.

Controlled release of IL-12 from hydrogel matrices at the resection site activates NK cells and CD8+ T cells locally while driving IFN-γ production that further reinforces M1 macrophage polarization. The spatially confined delivery of IL-12 is critical, as systemic IL-12 administration is dose-limited by severe hepatotoxicity and constitutional symptoms [107]. Hyaluronic acid hydrogels releasing a combination of GM-CSF and TLR-3 agonist poly(I:C) in a murine melanoma resection model demonstrated in situ dendritic cell recruitment and activation, with cross-priming of CD8+ T cells against tumor-associated antigens identified from the resected tumor material [108]. These locally primed T cells subsequently disseminated systemically, suppressing growth of pre-established lung metastases, demonstrating that the scope of therapeutic effect achievable by locally acting HNP platforms is not necessarily confined to the surgical site. The forward trajectory of this approach is represented by the combination of customized antigen loading in locally administered nanoparticulate vaccine formulations with neoantigen detection methodologies enabled by next-generation sequencing of resected tumor tissue [108].

## 7. Translational and Regulatory Challenges

### 7.1. Biocompatibility and Long-Term Safety

From preclinical proof-of-concept to clinical use, HNP composite platforms must overcome several challenging yet manageable translational hurdles. The most important of them is the need for thorough proof of biocompatibility across the variety of polymer compositions, nanoparticle types, and crosslinking chemistries used in these systems [109].

The foreign body response, which begins with protein adsorption to the material surface and culminates in macrophage-mediated encapsulation or destruction, controls inflammatory reactions to implanted biomaterials. In preclinical models, the majority of hydrogel systems, especially those based on PEG and hyaluronic acid, show little inflammatory responses and poor protein adsorption [110]. In any case, new factors are introduced by the nanoparticulate components. The increased intracellular delivery provided by cationic surface chemistry must be weighed against the charge-dependent toxicity of positively charged nanoparticles, which are associated with greater cytotoxicity and membrane disruption than negatively charged or neutral particles. The accumulation of inorganic nanoparticles, especially gold and iron oxide, in secondary organs like the liver and spleen has long-term effects on organ function that require careful assessment in chronic toxicity studies [111].

It is necessary to determine the biocompatibility of degradation by-products of both the polymer matrix and the nanoparticle shell. When PLGA breaks down into lactic and glycolic acid, the local pH drops, which can damage tissue and trigger an inflammatory reaction in high-loading conditions [112]. Case-by-case evaluation is necessary for the degradation products of polyurethane-based and some stimuli-responsive polymer systems. A dynamic toxicity profile, defined over time, is produced by the temporal evolution of material composition as degradation proceeds, with varying ratios of intact polymer, degradation intermediates, and end metabolites.

### 7.2. Manufacturing and Scalability

The difficulties in producing HNP composite systems are qualitatively different from those in producing simple polymer implants or traditional small-molecule drugs. These systems’ multi-component structure, which includes polymer matrices, nanoparticle dispersions, and one or more therapeutic payloads, increases the sources of variability and makes defining significant quality features more difficult [113,114].

Pharmacokinetics and biological performance are influenced by particle size, polydispersity, drug loading efficiency, and surface properties, all of which must be tightly controlled during nanoparticle manufacturing. For biologically derived components such as lipid nanoparticles and cell membrane-coated particles, whose biological raw materials introduce inherent variability, batch-to-batch reproducibility in particle production remains a persistent challenge [114]. The percentage of the input pharmaceutical product successfully incorporated into nanoparticles, or encapsulation efficiency, is sensitive to formulation conditions and needs to be optimized while minimizing the proportion of free drug that would not benefit from the controlled-release architecture.

Manufacturing becomes more complex when nanoparticles are incorporated into the hydrogel matrix. To achieve homogeneous particle dispersion without aggregation, it is necessary to adjust the mixing parameters, polymer concentration, and crosslinking process, while ensuring that the crosslinking chemistry does not compromise the integrity of the nanoparticles or the therapeutic payload. Process development that maintains consistency at higher volumes and flow rates is necessary to scale up from laboratory-scale synthesis to batch sizes compatible with clinical and commercial manufacturing. Sterilization is particularly difficult since the heat treatments or ionizing radiation used in traditional implants can denature protein payloads, break down polymer chains, or change the surface chemistry of nanoparticles. The two main practical options are aseptic processing in a cleanroom and ethylene oxide sterilization, both of which have significant consequences for cost and process complexity [115].

Despite their significant therapeutic potential, hybrid hydrogel-nanoparticle systems pose several challenges that must be considered when developing formulations. By necessitating precise control over nanoparticle production, batch-to-batch homogeneity, sterilization techniques, and homogeneous distribution within the hydrogel network, the addition of nanoparticles increases manufacturing complexity. Increased interactions with the polymer matrix may also result in prolonged nanoparticle retention, limiting their diffusion into adjacent tumor tissue and limiting intracellular drug delivery. Inadequate retention may also lead to early nanoparticle release and reduced localized therapeutic effect. Establishing the ideal balance between hydrogel architecture and nanoparticle mobility continues to be a crucial design factor. High nanoparticle loading may also make the hydrogel harder and more viscous, which could make it more difficult to manipulate and inject [84,116].

### 7.3. Regulatory Considerations

HNP composite platforms present a regulatory classification challenge that is not adequately addressed by existing frameworks designed for discrete drug, device, or biological product categories. As combination products, simultaneously incorporating a drug payload, a device scaffold, and in some cases a biological component such as a cell membrane coating or nucleic acid therapeutic, these systems fall under the competence of multiple regulatory centers and require a combination product pathway that must be designated by the FDA’s Office of Combination Products [117].

The regulatory submission for an HNP composite will typically require extensive characterization data to establish the identity, purity, and stability of each component and the integrated system. Non-clinical safety evaluation must demonstrate biocompatibility in accordance with ISO 10993, assess the biodistribution of nanoparticle components using appropriate analytical methodologies, and provide genotoxicity and carcinogenicity data for any degradation products with reactive functional groups. Pharmacology and pharmacokinetics data must characterize in vitro release kinetics and correlate them with in vivo pharmacokinetic profiles, establishing the drug concentration at the resection site and the systemic exposure fraction [118].

The specific, individualized character of many complex HNP platforms, along with the standardized evaluation methods required for regulatory approval, poses a challenge for clinical translation. Phase II clinical trials will provide initial efficacy signals and guide Phase III study design, whereas Phase I clinical trials will focus on safety and feasibility of surgical placement. The selection of appropriate clinical endpoints, local recurrence-free survival, overall survival, or pharmacodynamic biomarkers of target engagement, and the identification of patient populations most likely to benefit will be critical determinants of regulatory success [119].

### 7.4. Surgical Integration

The biological effectiveness of HNP composite platforms, as well as their smooth incorporation into surgical procedures, will ultimately determine their clinical significance. An injectable hydrogel system must be technically simple, take little time to apply during surgery, and work with typical neurosurgery, general surgical, or oncological surgical instruments and procedures [120].

Gelation kinetics must be calibrated to permit adequate time for cavity filling and positioning before setting. Rapid gelation is desirable to prevent liquid formulation from draining from open cavities, but premature gelation in the delivery syringe or catheter would be catastrophic. Injectable systems with gelation times in the range of 30–120 s under physiological conditions generally balance these competing requirements. The viscosity of the pre-gel solution determines the required needle gauge and injection pressure, with implications for tissue damage during administration. High-viscosity formulations may require large-bore needles incompatible with minimally invasive surgical approaches [121].

The optical and radiological properties of the hydrogel should ideally be compatible with intraoperative navigation systems. Hydrogels that are transparent or translucent under surgical lighting enable visual confirmation of complete cavity filling, while formulations compatible with intraoperative MRI or CT enable real-time verification of gel placement. Compatibility with drainage systems, wound closure techniques, and postoperative monitoring devices must be established to ensure that the HNP implant does not interfere with standard post-surgical care. Implementation rates in clinical practice will be influenced by the non-trivial challenge of surgeon education and trainingincluding knowledge of gel preparation methodology, injection technique, and recognition of adverse reaction events [122].

## 8. Emerging Trends and Future Directions

### 8.1. Smart Responsive Systems

In order to create systems that dynamically modify their activity in response to changing microenvironmental signals, the next generation of HNP platforms goes beyond passive stimuli-responsive release to include active sensing and adaptive therapeutic response capabilities. With carefully calibrated oxidative thresholds that differentiate the ROS levels of normal wound healing from those typical of the recurrent tumor niche, ROS-sensitive platforms using thioether, thioketal, and phenylboronic ester linkages are being developed. This allows for selective therapeutic activation only in the presence of pathological oxidative stress [123].

Enzyme-responsive polymer networks incorporating multiple cleavable substrates, combining MMP-sensitive crosslinks with cathepsin B-responsive nanoparticle surfaces, enable cascade release architectures in which the degradation of the hydrogel by one enzyme releases nanoparticles whose subsequent activation is triggered by a second enzyme upregulated in tumor cells’ intracellular environment [124]. Multi-stimuli-responsive platforms integrating pH, ROS, and enzymatic triggers exhibit gate-like behavior, requiring the simultaneous presence of multiple pathological signals to trigger drug release, thereby achieving a level of biological specificity that further minimizes off-target effects. Programmable degradation profiles achieved through spatially patterned crosslink density or hierarchical polymer architecture enable temporally structured release of multiple therapeutic agents, coordinating the delivery of different payloads to coincide with specific phases of the post-surgical biological response [124].

### 8.2. 3D Printed and Patient-Specific Hydrogels

Patient-specific anatomical variation in resection cavity geometry, particularly in glioblastoma and other CNS tumors, where cavities are complex, irregular, and adjacent to eloquent brain structures, motivates the development of patient-specific hydrogel implants that precisely conform to the resection void. Three-dimensional printing technologies, including extrusion-based bioprinting and digital light processing (DLP) photopolymerization, enable the fabrication of polymer scaffolds with geometries defined from intraoperative imaging data [124].

Pre-operative or intraoperative MRI data processed through image segmentation algorithms can generate 3D models of the anticipated resection cavity, which are then used to program the print path for a patient-specific hydrogel scaffold [124]. This scaffold can be pre-loaded with nanoparticle-drug composites during fabrication, with spatial variation in drug loading achievable by modulating nanoparticle concentration in the bioink. The functional integration of 3D-printed hydrogel implants with imaging-guided surgery, including real-time MRI or fluorescence-guidance, enables intraoperative verification of implant positioning and identification of residual tumor tissue adjacent to the implant boundary, thereby informing the distribution of therapeutic payload within the scaffold [125]. The technical requirements for intraoperative bioprinting are stringent, but advances in compact printing hardware and rapid photopolymerization chemistry are progressively reducing the barriers to implementation in the operating room.

### 8.3. Personalized Local Therapeutic Platforms

The fundamental premise of personalized medicine, that therapeutic strategies tailored to the molecular and immunological characteristics of an individual patient’s tumor will outperform population-average treatments, applies with particular force to the design of local post-surgical platforms. The molecular profiling of resected tumor tissue, now routinely performed as part of standard oncological workup in most cancer centers, generates a rich dataset encompassing mutational landscape, copy number alterations, transcriptomic profiles, and immunohistochemical receptor expression patterns [126].

Matching the therapeutic payload to the unique molecular vulnerabilities of each patient’s residual tumor is made possible by biomarker-guided therapy selection for loading into the HNP platform. While a patient with high HER2 expression might receive a trastuzumab-functionalized nanoparticle component in addition to a traditional cytotoxic payload, a patient whose tumor expresses high PD-L1 and is infiltrated by exhausted T cells would benefit from a locally acting checkpoint inhibitor component [127]. Further optimization of drug release patterns would be possible with precision dosing strategies that account for patient-specific pharmacokinetic characteristics, such as local tissue perfusion, cavity capacity, and expected hydrogel disintegration rates based on inflammatory biomarkers. By integrating molecular recognition components that detect patient-specific biomarkers in the tumor microenvironment and adjust drug release appropriately, the adaptive material concept goes beyond customization [128]. While largely in the research phase, these approaches are progressing rapidly and may constitute standard elements of HNP platform design within the coming decade.

### 8.4. Integration with Advanced Immunotherapies

The convergence of locally acting HNP platforms with the most powerful systemic immunotherapies, including adoptive cell therapies and mRNA-based modalities, represents the frontier of combination oncological treatment strategies. CAR-T cell therapy, which has transformed the treatment of certain hematological malignancies, faces significant barriers to efficacy in solid tumors, including inadequate T cell infiltration, immunosuppressive TME, antigen heterogeneity, and T cell exhaustion [129].

Local delivery of CAR-T cells or cytokines and checkpoint inhibitors that support CAR-T cell persistence and functionithin HNP composite platforms addresses the trafficking and microenvironmental barriers to solid tumor CAR-T efficacy [130]. Preclinical studies in glioblastoma models have demonstrated that hyaluronic acid hydrogels releasing CAR-T cells targeting EGFRvIII achieve superior local tumor control compared to systemically infused CAR-T cells, with evidence of sustained T cell persistence and reduced T cell exhaustion attributable to the niche-like support provided by the hydrogel matrix. mRNA-based immunotherapies delivered via lipid nanoparticles embedded in hydrogel matrices enable local expression of tumor-associated antigens, immune stimulatory co-receptors, or reprogramming factors that convert immunosuppressive cells into immune effectors [131]. A reversible, controlled therapeutic intervention that can be adapted to the temporal dynamics of the post-surgical immune response is enabled by the temporal expression profile of mRNA payloads. Further biological sophistication that leverages natural cell trafficking and homing behaviors to target residual tumor cells is provided by cell-based delivery systems, such as tumor-derived exosomes engineered to carry therapeutic payloads or mesenchymal stem cells loaded with cytotoxic nanoparticles, both implemented within hydrogel matrices [130,131].

## 9. Conclusions

From initial conceptual displays to a mechanistically advanced, preclinically validated therapeutic approach, the field of hydrogel-nanoparticle composite systems for post-surgical cancer therapy has evolved. Several key conclusions about the design principles, mechanistic advantages, and translational potential of these systems are provided by the evidence reviewed in this paper.

From an architectural perspective, the combination of a conformable, biodegradable hydrogel matrix with stimuli-responsive nanoparticle carriers creates a dual-level controlled release system whose pharmacological versatility exceeds that of either component alone [132]. The ability to deliver multiple therapeutic agents, cytotoxic drugs, immunomodulatory biologics, gene therapy payloads, and physically acting modalities such as photothermal therapy, within a single implantable composite provides a multimodal approach to recurrence prevention that addresses the mechanistic complexity of residual disease. The stimuli-responsive properties of advanced HNP systems, linking drug release to pathological signals specific to the post-surgical tumor microenvironment, represent a significant advance in the selectivity of local therapy.

The mechanistic evidence for immunomodulation as a central component of HNP platform efficacy is particularly compelling. The conversion of the immunosuppressive post-surgical niche into an immunostimulatory environment through local delivery of checkpoint inhibitors, TLR agonists, and pro-inflammatory cytokines, with evidence of systemic anti-tumor immune activation extending beyond the resection site, transforms locally acting platforms from pure drug-delivery devices into initiators of systemic immune responses [133]. If demonstrated in clinical settings, this systemic feature of local therapy would significantly enhance the therapeutic impact of locally injected HNP platforms.

The translational pathway for HNP systems is demanding, requiring integration of biomaterial science, pharmaceutical development, immunology, and surgical expertise within a regulatory framework that is still evolving to accommodate combination products of this complexity. Nevertheless, the convergence of enabling technologies, including advanced polymer chemistry, scalable nanoparticle manufacturing platforms validated for clinical use in mRNA vaccines, and increasingly sophisticated intraoperative imaging and surgical navigation, creates a favorable environment for clinical translation. Injectable hydrogel systems for GBM and breast cancer are currently undergoing first-in-human trials; the collection of clinical pharmacodynamic and safety data from these trials will be crucial in improving the design criteria for next-generation platforms [134].

The development of 3D-printed, imaging-guided implants adjusted to specific resection cavity geometries, the combination of HNP systems with newly developed cell-based and nucleic acid immunotherapies, and the integration of molecular tumor profiling with patient-specific platform design all point to a study plan with significant ambition and clinical promise. The scientific evidence reviewed here provides a strong basis for exploring this therapeutic approach, but realizing this promise will require ongoing interdisciplinary collaboration and investment.

## Figures and Tables

**Table 1:** Representative preclinical studies of injectable hydrogel-nanoparticle platforms for preventing postoperative tumor recurrence [99,100,101,102,103].

Hydrogel Matrix	NP Composition/Core-Shell Chemistry	Loaded Therapeutics	Experimental Model	Post-Surgical Resection Model	Key Survival/Recurrence Metrics	Reference
Injectable hydrogel formed by mixing a thrombin solution, a fibrinogen solution containing ATRA and a MN/NO-based immune nano-activator	MPB-NO@DOX NPs: mesoporous Prussian Blue core loaded with doxorubicin and coated with MNO2 shell	DOX + ATRA + NO	Murine postoperative tumor resection model	Yes	The treatment significantly reduced postoperative tumor recurrence, producing the lowest recurrence rate	[103]
Injectable GelMA hydrogel	ZIF-8 metal-organic framework NPs coated with CO_2_ NPS	DOX	Orthotopic 4T1 breast cancer postoperative model	Yes	The treatment significantly inhibited postoperative tumor reccurence, resulting in the lowest tumor volume and weight. The hydrogel also promoted rapid wound healing (aprox. 90% wound closure after 12 days)	[102]
Injectable autocatalytic hydrogel based on oxidized dextran and gelatin	Cooper peroxide NPs dispersed throughout the hydrogel	Without classic therapeutics. Copper peroxide NPs themselves act as ROS generators	Orthotopic 4T1 breast cancer postoperative model	Yes	The treatment significantly reduced postoperative tumor recurrence, inducing pyroptosis.	[101]
Injectable thermosensitive hydrogel based on PLGA-Peg-PLGA triblock copolymer	Carbon nitride nanosheets coated with single-atom platinum nanozyme (CN-Pt)	Gemcitabine + CN-Pt + NIR irradiation (photothermal irradiation)	Incomplete postoperative Lewis lung carcinoma mouse model	Yes	The treatment significantly reduced postoperative tumor recurrence, with 4/6 mice showing 60% survival for at least 55 days. Also, treatment showed nearly complete antibacterial activity after NIR irradiation and accelerated wound healing.	[100]
Injectable thermosensitive PEG hydrogel	Dendritic mesoporous silica NPs loaded with glucose oxidase and functionalized with CU2+ ions	Glucose oxidase and CU2+ for enzyme dynamic therapy and ROS generation	Postoperative T-24 bladder cancer recurrence model	Yes	The treatment significantly reduced postoperative tumor recurrence; all treated mice remained free of postoperative tumor regrowth, whereas surgery alone resulted in marker tumor recurrence.	[99]

## Data Availability

No new data were created or analyzed in this study. Data sharing is not applicable to this article.

## Abbreviations

The following abbreviations are used in this manuscript:

HNP	Hydrogel-nanoparticle
TME	Tumor microenvironment
GBM	Glioblastoma multiforme
MRD	Minimal residual disease
BBB	Blood-brain barrier
DAMPs	Damage-associated molecular patterns
MMPs	Matrix metallo-proteinases
HIF-1α	Hypoxia-inducible factor 1-alpha
ROS	Reactive oxygen species
CSCs	Cancer stem cells
EMT	Epithelial-mesenchymal transition
MDSCs	Myeloid-derived suppressor cells
PEG	Polyethylene glycol
PNIPAM	Poly(N-isopropylacrylamide)
LCST	Lower critical solution temperature
EPR	Enhanced permeability and retention
PLGA	Poly(lactic-co-glycolic acid)
SLNs	Solid lipid nanoparticles
MSNs	Mesoporous silica nanoparticles
IONPs	Iron oxide nanoparticles
TLR	Toll-like receptor
ICD	Immunogenic cell death
TAMs	Tumor-associated macrophages
DLP	Digital light processing
